# Continuation of immunosuppression vs. immunosuppression weaning in potential repeat kidney transplant candidates: a care management perspective

**DOI:** 10.3389/fneph.2023.1163581

**Published:** 2023-06-07

**Authors:** Michelle J. Hickey, Gurbir Singh, Erik L. Lum

**Affiliations:** ^1^ Department of Pathology and Laboratory Medicine, University of California, Los Angeles (UCLA) Immunogenetics Center, David Geffen School of Medicine, Los Angeles, CA, United States; ^2^ Department of Medicine, Division of Nephrology, University of California, Los Angeles (UCLA) David Geffen School of Medicine, Los Angeles, CA, United States

**Keywords:** kidney, immunosuppression withdrawal, failed allograft, allosensitization, transplant

## Abstract

Management of immunosuppression in patients with a failing or failed kidney transplant requires a complete assessment of their clinical condition. One of the major considerations in determining immunosuppression is whether or not such an individual is considered a candidate for re-transplantation. Withdrawal of immunosuppression in a re-transplant candidate can result in allosensitization and markedly reduce the chances of a repeat transplant. In this review, we summarize the effects of immunosuppression reduction on HLA sensitization, discuss the impacts of allosensitization in these patients, and explore reduction protocols and future directions. Risks of chronic immunosuppression, medical management of the failing allograft, and the effect of nephrectomy are covered elsewhere in this issue.

## Introduction

Kidney transplantation is the optimal treatment for individuals with end-stage kidney disease (ESKD). The recipients of kidney transplants experience significant increases in life expectancy and quality of life, and there are reductions in the overall societal cost of ESKD care (1–3). The success of transplant is the result of the development of effective immunosuppression (4, 5).

However, long-term allograft survival has remained unchanged over the past several decades, with 10-year graft survival around 40%–50% (5). Exposure to chronic immunosuppression may partially be responsible for this phenomenon. It has been well established that immunosuppressive medications used in transplantations increase the risks of diabetes, malignancy, and infection, and result in premature allograft loss (6, 7). Calcineurin inhibitors (CNIs), which make up over 90% of immunosuppressive regimens, have inherent renal toxicity (8–10). Furthermore, current immunosuppressive medications appear to have little protection against chronic rejection, a leading cause of graft failure (11). The end result is a return to dialysis for many patients with a transplant.

It is estimated that approximately 4% of prevalent dialysis patients in the United States are prior kidney transplant recipients (12). Studies demonstrate that for transplant recipients who return to dialysis, there are increased risks of cardiovascular complications, infection, and death compared with incident dialysis patients without a transplant (13–15). One plausible explanation for these observations is the continued use of immunosuppressive medications, which increase the risk of infections and lead to an adverse cardiovascular profile (13). Prior kidney transplant recipients who return to dialysis may also represent a sicker population, returning to dialysis after medical complications. Despite the increased mortality rates in patients who are on dialysis re-transplantation remains a realistic option, as approximately 15% of the deceased donor kidney waiting list in the United States is composed of prior transplant recipients (16). The ongoing use of immunosuppressive medications may alleviate the consequences of chronic inflammation of the allograft, preserve residual renal function, and reduce allosensitization to improve re-transplant opportunities.

This review will focus on kidney transplant recipients with failing allografts who remain potential re-transplant candidates, the effects of immunosuppression changes on allosensitization and re-transplantation in these candidates, current recommendations, and areas of future exploration. The risks of continued immunosuppression, the role of immunosuppression reduction, management of a failing kidney allograft and initiation of renal replacement therapy, and nephrectomy are covered elsewhere in this issue.

## Immunosuppression in kidney transplant recipients

The development of effective immunosuppressive medications has resulted in a dramatic increase in allograft survival since the first successful transplant in 1954. Exposure to these medications results in an immunological state of acceptance, which requires the ongoing use of immunosuppressive medications to prevent the normal host reaction against the allograft (17). Discontinuation, or significant disruptions in medication dosing leading to subtherapeutic exposure, results in the restoration of normal host immune responses and rejection of the allograft.

Rejection of a kidney transplant is broadly divided into two categories, acute and chronic, which are further subdivided into T-cell-mediated rejection (TCMR) and antibody-mediated rejection (ABMR). Although a review of rejection is beyond the scope of this discussion, it is sufficient to highlight that rejection is a leading cause of allograft impairment and allograft failure with a return to dialysis (18). Rejection episodes may have profound impacts on kidney transplant recipients, resulting in severe allograft impairment, premature allograft loss, and/or significant allosensitization (19). A recent publication from Australia and New Zealand demonstrated that there are significant long-term complications associated with even a single rejection episode, including increased long-term risks of chronic allograft injury, cardiovascular disease, infection, and cancer (20).

ABMR, in particular, carries a poor prognosis, with graft survival substantially lower than that of TCMR (21). ABMR is a B-cell phenomenon that results in the development of anti- human leukocyte antigen (HLA) antibodies against the allograft (22). These antibodies are specifically targeted against the allograft and result in the activation of the complement system, immune cell activation and maturation, cellular infiltration into the allograft, and endothelial cell changes resulting in tissue injury. One of the difficulties in managing ABMR is that the therapies do not effectively eliminate B-cell clones, the cells primarily responsible for antibody production, resulting in persistent anti-HLA antibodies and injuries despite treatment (23). Donor-specific anti-HLA antibodies (DSAs) are a barrier to re-transplantation as exposure to these “repeat mismatches” results in a robust response and reduced allograft survival (24). This process can be further compounded by the development of additional anti-HLA antibodies specific to antigens that are not displayed by the allograft, resulting in a broad sensitization that can further limit future transplant options for these individuals. Kidney transplant candidates who are highly sensitized have historically had decreased rates of deceased donor transplantation (25, 26).

## Impact of immunosuppression withdrawal in patients with a failing allograft

As highlighted in the previous section, reductions in immunosuppression can result in increased allosensitization in patients with a functioning kidney allograft. It should stand to reason that patients with a failed allograft undergoing immunosuppression reduction should also carry these risks. Several single-center retrospective studies have demonstrated a marked increase in anti-HLA antibody formation in prior transplant recipients who undergo immunosuppression reduction and withdrawal (27–32).

In a single-center study, Del Bello and colleagues analyzed the formation of DSAs in individuals with failed allografts who underwent immunosuppression withdrawal, with and without nephrectomy (29). Of the 21 patients who did not undergo allograft nephrectomy, six had HLA-DSAs at the time of graft failure. Following immunosuppression withdrawal, this number increased to 11 patients (51%). The analysis demonstrated a much larger impact on class II DSA formation than on class I (43% vs. 24%).

DSA formation increases with the progressive weaning of immunosuppression. Nimmo et al. analyzed 17 patients who underwent immunosuppression withdrawal and underwent DSA testing pre, during, and post immunosuppression withdrawal to determine the temporal pattern of DSA formation (27). DSA formation increased from 13% pre-immunosuppression weaning to 40% during weaning and 62% following immunosuppression withdrawal. It was estimated that this increase in sensitization would result in a reduction in 5-year transplantation rates from 54% to 46%.

Anti-HLA antibody formation during immunosuppression weaning is not just limited to DSAs. Immunosuppression withdrawal in patients with allograft failure also leads to the production of anti-HLA antibodies directed against a broad range of HLA antigens, hence reducing the potential donor pool available for the patient. Augustine et al. examined the outcomes of 300 consecutive patients with allograft failure (28). Twenty-one percent of patients were highly sensitized, defined as a class I or II, or having a panel-reactive antibody (PRA) of > 80% at the time of allograft failure, which increased to 68% at a time point of 24 months following graft failure. This effect was particularly profound in the 119 patients with a low PRA before transplant, with 56% becoming highly sensitized during the study. Notably, the increase in sensitization was seen only in the group of patients who underwent weaning of immunosuppression, as those who were maintained on CNI therapy did not develop DSAs.

Scornik et al. (30) also showed the benefit of continued immunosuppression in reducing allosensitization. In this study of 104 patients with allograft failure, those who were maintained on immunosuppression did not develop anti-HLA antibodies. Individuals in this study who discontinued immunosuppression went on to become sensitized, even in the absence of transfusion or allograft nephrectomy, both of which are considered sensitizing events. The overall frequency of sensitization in this group was 70%, despite over 81% of transplant recipients not having anti-HLA antibodies at the time of allograft loss, suggesting that continued immunosuppression is essential for preventing anti-HLA antibody formation following allograft failure and during potential sensitizing events. These results indicate a clear benefit in reducing allosensitization with the continuation of immunosuppression in re-transplant candidates with a failed allograft. A summary of these studies is shown in **Table 1**.

**Table 1:** Summary of major studies in immunosuppression withdrawal in failed kidney transplant recipients.

Study	IS protocol	Measurements of allosensitization
Del Bello et al. (29)	Single-center patients with a failed kidney transplant who underwent complete immunosuppression withdrawal 69 total patients (48 nephrectomy, 21 *in situ)*	Allograft nephrectomy group DSA: 12.5% at baseline, > 81% post nephrectomy Non-allograft nephrectomy group DSA: 14.2% at baseline, > 52.4% Overall rate of class I HLA ab 23% non-nephrectomy, 77% nephrectomy Overall rate of class II HLA Ab 42.8% non-nephrectomy, 62.5% nephrectomy
Nimmo et al. (27)	Single-center study looking at DSA formation rates in patients undergoing IS withdrawal 41 total patients (24 nephrectomy, 17 *in situ)*	Increase in DSAs from 13% pre weaning, to 40% post weaning, to 62% post withdrawal
Augustine et al. (28)	Single-center examination of 300 consecutive patients with failed kidney allograft 119 patients with low PRA prior to transplant analyzed	Percentage of patients who were highly sensitized (PRA > 80%) increased from 20% at failure to 68% post weaning
Scornik et al. (30)	Single-center evaluation of 104 patients	19% developed DSA prior to graft loss with increase to 55% post immunosuppression weaning

An additional complication of immunosuppression withdrawal in patients with a failed allograft is graft intolerance syndrome. This condition is characterized by inflammation of the allograft with fever, gross hematuria, and/or graft tenderness, and is the leading indication of transplant allograft nephrectomy following graft failure (33). Transplant nephrectomy is associated with an increase in allosensitization, with some studies estimating the *de novo* DSA formation in up to 80% of individuals with a failed transplant requiring allograft nephrectomy (27–29, 32). The exact reasons for increased allosensitization remain unclear but may be related to immunological events precipitating allograft intolerance, reductions in immunosuppression, the allograft acting as a sink for circulating anti-HLA antibodies, and an increase in the rate of blood transfusions in this setting (34).

## Immunosuppression reduction: who to consider?

Following allograft failure and return to dialysis, the decision to discontinue immunosuppression in a prior transplant recipient should be taken with careful consideration of the individual’s clinical status and potential for re-transplantation. Individuals for whom transplantation is unlikely to occur or with life-threatening complications of immunosuppression should undergo reductions in immunosuppression regardless of the risks of rejection and sensitization. Withdrawing immunosuppression on dialysis may reduce the mortality of patients awaiting transplantation, suggesting that continued immunosuppression to avoid sensitization must be balanced against the risks to the patient. In a single-center study, Ryu et al. demonstrated that the continuation of immunosuppression was associated with a three-fold increase in mortality compared with weaning immunosuppression in individuals experiencing allograft failure (30). This reduction in mortality was driven by a reduction in infectious and cardiovascular complications, two of the most common causes of death on dialysis.

In individuals without complications from immunosuppression and who are expected to undergo re-transplantation soon after experiencing graft failure, continuing immunosuppression, especially CNIs, to avoid allosensitization is advised (35). The increase in sensitization following immunosuppression withdrawal may result in significant changes in a candidate’s waitlist position and continued immunosuppression may result in reduced allosensitization (36). Thus, the decision to continue, reduce, or withdraw immunosuppression in a patient with a failed kidney allograft must be individualized. It is currently recommended that patients with an anticipated wait for re-transplantation of over 1 year be considered for a reduction in immunosuppression, as the risks of ongoing immunosuppression are felt to outweigh the benefits of avoiding sensitization for a transplant that is not to occur for some time.

## How to reduce immunosuppression in a patient with a failed allograft

Currently, there is sparse evidence on the degree and rate of immunosuppression reduction for patients who are awaiting re-transplantation. Surveys of transplant centers indicate that most centers begin with the removal of the anti-metabolite, followed by either CNI or prednisone reduction over an unspecified period of time (37, 38). Over 20% of those surveyed reported no defined protocol for withdrawal (37, 38). The single most important factor in deciding on the continuation of immunosuppression was whether or not a patient was re-listed for a kidney transplant (37, 38).

Rapid immunosuppression withdrawal results in an increased risk of allosensitization and loss of residual renal function rather than a prolonged withdrawal. In a single-center study, Casey et al. analyzed the effects of the rate of immunosuppression withdrawal in 49 patients experiencing graft failure (39). Approximately 60% of patients who underwent a long taper remained unsensitized, compared with 30% for the faster taper, resulting in an odds ratio of 5.8 favoring prolonged immunosuppression withdrawal for avoiding sensitization.

A variety of complex protocols exist depending on the timing of graft failure post transplant, the presence of concurrent infection or malignancy in the patient, re-transplant likelihood, and residual renal function. Given the unique individual needs of each patient, a one-size-fits-all protocol is unlikely to be developed. However, we can take the lessons learned from transplant recipients with a functioning graft and apply them to this population.

The most common maintenance immunosuppression regimen post-kidney transplantation utilizes a combination of corticosteroids, CNIs, and anti-metabolites. In clinical practice, the anti-metabolite is the most commonly removed medication in settings of infection or malignancy, with low associated rates of near-term allograft loss. In a single-center study looking at the removal of mycophenolate mofetil for clinical indications, Park et al. demonstrated that death-censored 1-year graft survival was only slightly lower than for those who remained on the medication (40). There was an increased risk of rejection in the MMF withdrawal group (27.4% vs. 8.9%), and a significant reduction in graft survival after 1 year for the mycophenolate removal group, but this may have been confounded for medication withdrawal as patient survival was significantly lower in the MMF withdrawal group.

Late steroid withdrawal in stable kidney transplant recipients is associated with a > 30% increased risk of acute rejection, compared with steroid maintenance (41). In a meta-analysis by Ali et al., 1,907 patients were analyzed for the effect of late steroid withdrawal (41). Late withdrawal was associated with a 34% increased risk of rejection, but also a 35% and 5% reduction in overall graft failure and all-cause mortality, respectively. Pediatric growth outcomes and total cholesterol levels were also markedly improved with steroid reduction. Therefore, although there may be an increased risk of rejection with late steroid withdrawal, in a patient who already has a failed allograft, there may be additional health cardiovascular benefits to removing prednisone.

Calcineurin inhibitors have become the backbone of the immunosuppression regimen in solid organ transplantation since their introduction in the early 1980s. Despite the myriad of downsides to their use, they provide the best options for preventing rejection. The Clinical Trials in Organ Transplantation (CTOT)-9, which studied the effects of tacrolimus withdrawal in non-sensitized living donor kidney transplant recipients randomized 21 patients to either continued maintenance immunosuppression or tacrolimus withdrawal (42). Recipients received rabbit anti-thymocyte globulin and were maintained on tacrolimus, MMF, and prednisone, and at 6 months participants without DSA, rejection, or inflammation on a protocol biopsy were randomized to wean off or remain on tacrolimus. The study was terminated prematurely because of unacceptable rates of acute rejection and/or development of *de novo* DSA. The impact of CNI discontinuation on allosensitization in a failed allograft was demonstrated in the aforementioned Augustine study in which the rate of becoming highly sensitized increased from 21% to 68% during immunosuppression withdrawal for those not maintained on CNI (28). Combined, these studies suggest that continued low-dose CNI use may be superior for avoiding allosensitization in kidney transplant recipients with allograft failure.

One emerging option is to switch from a CNI to belatacept. Belatacept is a costimulatory inhibitor that, on average, increases the estimated glomerular filtration rate (eGFR) by 10–12 mL/minute/1.73 m^2^ compared with CNI therapy and has the added benefit of a reduction in *de novo* DSA formation (43). In a randomized study by Budde et al., conversion from a CNI to belatacept-based maintenance immunosuppression resulted in an increase in eGFR, from 48.5 mL/minute/1.73 m^2^ to 55.5 mL/minute/1.73 m^2^ after 24 months, with similar rates of adverse events and a marked reduction in DSA formation: 1% for the belatacept group compared with 7% for the CNI group. Interestingly, this study showed a greater improvement in eGFR with a lower initial eGFR when converting from CNI to belatacept, suggesting a role in the failing allograft. Conversion may permit prolonged graft survival and increase the chances of a pre-emptive transplant. However, belatacept use is associated with a higher risk of cellular rejection and carries an increased risk of central nervous system post-transplant lymphoproliferative disease, especially in individuals who are Epstein–Barr virus (EBV) seronegative, and viremias (44). This option should be considered in suitable individuals with a failing allograft not yet on dialysis to prolong allograft survival and are EBV seropositive; however, once renal replacement therapy has been initiated the benefit is unclear.

Given the available limited data, discontinuation of the anti-metabolite is a reasonable first step in a kidney transplant recipient with a failed allograft. Whether or not this should occur at the time of renal replacement therapy is unclear. A slow weaning of steroids and/or CNIs over > 3 months is recommended over a more rapid taper; whether this should be done in sequence or in tandem is unknown. Current guidelines recommend that CNIs are reduced by 50% every 3 months, with consideration to stop prednisone at any time or remain on it indefinitely. This recommendation seems reasonable given the importance of CNIs in preventing DSA formation and the effects of prednisone on CV risk. More studies are needed to determine the order and timing of immunosuppression withdrawal, optimal if any immunosuppression, and the temporal effects on allosensitization.

## Monitoring in patients with graft failure while undergoing immunosuppression reduction

Surveillance anti-HLA antibody monitoring during immunosuppression weaning may enable the early detection of allosensitization and provide a signal to maintain immunosuppression in a clinically stable patient with allograft failure. However, the logistics of testing remain challenging. Often the patient with a failed allograft is no longer under the care of the transplant center and the transplant team may be unaware of changes in immunosuppression. Often these changes are performed under the care of the local treating nephrologist. An additional challenge is that once patients become broadly sensitized, treatments to desensitize patients to improve transplant likelihood are ineffective (45).

It is required that anti-HLA antibody testing by solid-phase methods, such as single-antigen bead testing, be obtained prior to active re-listing for repeat kidney transplant, and screening is recommended while awaiting transplantation. However, the ideal frequency of testing is unknown. From the results of this testing, unacceptable antigens can be entered into UNET, and data are available for virtual and physical crossmatch interpretations. Longitudinal assessment is critical for determining if a patient’s antibody strengths and specificities are ‘stable’ after immunosuppression is removed. In 2014, the kidney allocation system (KAS) was changed to increase the priority for sensitized patients. The implementation of the KAS resulted in a system that prioritizes higher levels of sensitization. The effects of this resulted in a period of a marked increase in the transplantation rate in waitlist candidates with a PRA > 80%, significantly shortening their waiting times (45–47). Patients with high calculated panel-reactive antibodies (cPRAs) may receive donor offers quickly if they have a large number of allocation points and the benefit of common haplotypes. If a recent expansion of anti-HLA antibodies is identified during the virtual crossmatch and risk assessment, then there may be a need to draw a new sample urgently for solid-phase testing or a crossmatch to make decisions on organ acceptance. Additional solid-phase testing with serum dilution or with the C1q modification of the test (48, 49) may be necessary to identify antibodies that are “pro-zoned” or in excess, and, therefore, appear inaccurately weak (50, 51).

It is clear that improved monitoring before anti-HLA antibody formation occurs is necessary to determine the effects of immunosuppression weaning in patients with a failed allograft. Future studies are necessary to determine the optimal frequency of anti-HLA antibody testing, the kinetics of anti-HLA antibody formation in patients with a failed allograft undergoing immunosuppression withdrawal, and if testing will improve transplant outcomes.

Donor-derived cell-free DNA assays have been shown to reliably detect the presence of antibody-mediated rejection in kidney transplant recipients with an elevated creatinine level, especially if concurrent DSA is present. More recently, the ADMIRAL study demonstrated the potential efficacy of surveillance Donor-derived cell-free DNA (dd-cfDNA) testing (52). In this study, the presence of persistent elevations in cf-ddDNA was associated with an increased risk of allograft loss and preceded the development of DSA by 30 days. Further studies are needed to validate the use of dd-cfDNA as a surveillance tool for immune quiescence and its role in patients awaiting kidney transplantation with a failed allograft. However, its potential use in the surveillance of a failed allograft undergoing immunosuppression withdrawal may provide vital information on immune reactivity and allow for the personalization of immunosuppression withdrawal in clinically stable patients to minimize the risks of allosensitization.

## Conclusion

The withdrawal of immunosuppression in patients with a failed kidney transplant results in an increased risk of allosensitization. Current guidelines recommend tapering immunosuppression if patients are not re-transplant candidates, and for re-transplant candidates who have an expected waiting time of > 1 year to re-transplant due to an increased risk of complications associated with ongoing chronic immunosuppression. Longitudinal anti-HLA antibody testing with solid-phase methods is required for re-listing for repeat organ transplantation and periodic testing is recommended while awaiting transplantation. Current practices vary significantly, and clinical trials are needed to determine how best to reduce immunosuppression and provide immunosurveillance in patients who are awaiting re-transplantation.

## Author contributions

EL was responsible for initial draft and edits. MH was responsible for the initial draft of the section entitled “Monitoring in patients with graft failure while undergoing immunosuppression reduction” and edits. GS provided edits, drafted table and wrote the reference section. All authors contributed to the article and approved the submitted version.

## References

[1] Wolfe RA Ashby VB Mildford EL. Comparison of mortality in all patients on dialysis, patients on dialysis awaiting transplantation, and recipients of a first cadaveric transplant. N Engl J Med (1999) 341:1725–30. doi: 10.1056/NEJM199912023412303 10580071

[2] Masud Iqbal M Rahman N Alam M Deb Nath PK Waheed S Islam K. Quality of life is improved in renal transplant recipients versus that shown in patients with chronic kidney disease with or without dialysis. Exp Clin Transpl (2020) 18(Suppl 1):64–7. doi: 10.6002/ect.TOND-TDTD2019.P11 32008498

[3] Fu R Sekercioglu N Berta W Coyte PC. Cost-effectiveness of deceased-donor renal transplant versus dialysis to treat end-stage renal disease: a systematic review. Trans Direct (2020) 6(2):e522. doi: 10.1097/TXD.0000000000000974 PMC7004633 32095508

[4] Wang JH Skeans MA Israni AK. Current status of kidney transplant outcomes: dying to survive. Adv Chronic Kidney Dis (2016) 23(5):281–6. doi: 10.1053/j.ackd.2016.07.001 27742381

[5] Harihan Israni AK Danovitch G. Long-term survival after kidney transplantation. N Engl J Med (2021) 385:729–43. doi: 10.1056/NEJMra2014530 34407344

[6] Wojciechowski D Wiseman A. Long-term immunosuppression management. Clin J Am Soc Neph (2021) 16(8):1264–71. doi: 10.2215/CJN.15040920 PMC8455033 33853841

[7] Agrawal A Ison MG Danziger-Isakov L. Long-term infectious complications of kidney transplantation. Clin J Am Soc Neph (2022) 17(2):286–95. doi: 10.2215/CJN.15971020 PMC8823942 33879502

[8] Farouk S Rein JL. The many faces of calcineurin inhibitor toxicity – what the FK? Adv Chronic Kidney Dis (2020) 27(1):56–66. doi: 10.1053/j.ackd.2019.08.006 32147003 PMC7080294

[9] Ojo AO Held PJ Pork FK Wolfe RA Leichtman AB Young EW. Chronic renal failure after transplantaiton of a nonrenal organ. N Engl J Med (2003) 349:931–40. doi: 10.1056/NEJMoa021744 12954741

[10] Naesens M Kuypers DR Sarwal M. Calcineurin inhibitor nephrotoxicity. Clin J Am Soc Neph (2009) 4(2):481–508. doi: 10.2215/CJN.04800908 19218475

[11] Lai X Zheng X Mathew JM Gallon L Leventhal JR Zhang ZJ. Tackling chronic kidney transplant rejection: challenges and promises. Front Immunol (2021) 12:661643. doi: 10.3389/fimmu.2021.661643 34093552 PMC8173220

[12] Fiorentino M Gallo P Giliberti M Colucci V Schena A Stallone G. Management of patients with a failed kidney transplant: what should we do? Clin Kid J (2021) 4(1):98–106. doi: 10.1093/ckj/sfaa094 PMC7857798 33564409

[13] Requiao-Moura LR Moreira Albino CR Rebello Bicalho P Ferraz ÉA Pires LMMB da Silva MFR. Long-term outcomes after kidney transplant failure and variables related to risk of death and probability of retransplant: results from a single-center cohort study in Brazil. PloS One (2021) 16(1):e0245628. doi: 10.1371/journal.pone.0245628 33471845 PMC7816974

[14] Davis S Mohan S. Managing patients with failing kidney allograft. Clin J Am Soc nephrol (2022) 17:444–51. doi: 10.2215/CJN.14620920 PMC8975040 33692118

[15] Pham PT Everly M Faravardeh Pham PC. Management of patients with a failed kidney transplant: dialysis reinitiation, immunosuppression weaning, and transplantectomy. World J Nephrol (2015) 4(2):148–59. doi: 10.5527/wjn.v4.i2.148 PMC4419125 25949929

[16] Clark S Kadatz M Gill J Gill JS. Access to kidney transplantation after a failed first kidney transplant and associations with patient and allograft survival. Clin J Am Soc Neph (2019) 14(8):1228–37. doi: 10.2215/CJN.01530219 PMC6682813 31337621

[17] Slepicka PF Yazdanifar M Bertaina A. Harnessing mechanisms of immune tolerance to improve outcomes in solid organ transplantation: a review. Front Immunol (2021) 12:688460. doi: 10.3389/fimmu.2021.688460 34177941 PMC8222735

[18] Sellares J de Frietas DG Mengel M Reeve J Einecke G Sis B. Understanding the causes of kidney transplant failure: the dominant role of antibody-mediated rejection and nonadherence. Am J Trans (2012) 12(2):388–99. doi: 10.1111/j.1600-6143.2011.03840.x 22081892

[19] Lionaki S Panagiotellis K Iniotaki A Voletis JN. Incidence and clinical significance of de Novo donor specific antibodies after kidney transplantation. Clin Dev Immunol (2013) 2013:849835. doi: 10.1155/2013/849835 24348683 PMC3856119

[20] Clayton PA McDonald SP Russ GR Chadban SJ. Long-term outcomes after acute rejection in kidney transplant recipients: an ANZDATA analysis. J Am Soc Nephrol (2019) 30(9):1697–707. doi: 10.1681/ASN.2018111101 PMC6727270 31308074

[21] Carlos Garces J Guisti S Staffeld-Coit C Bohorquez H Cohen AJ Loss GE. Antibody-mediated rejection: a review. Oschner J (2017) 17(1):46–55. PMC5349636 28331448

[22] Thomas Valenzuela NM Reed E. The perfect storm: HLA antibodies, complement, FcγRs and endothelium in transplant rejection. Trends Mol Med (2015) 21(5):319–29. doi: 10.1016/j.molmed.2015.02.004 PMC4424065 25801125

[23] Budde K Burr M. Any progress in the treatment of antibody-mediated rejection? J Am Soc Nephrol (2018) 29(2):350–2. doi: 10.1681/ASN.2017121296 PMC5791083 29371423

[24] Lefaucher C Loupy A Hill GS Andrade J Nochy D Antoine C. Preexisting donor-specific HLA antibodies predicy outcome in kidney transplantation. J Am Soc Nephrol (2010) 21(8):1398–406. doi: 10.1681/ASN.2009101065 PMC2938596 20634297

[25] Bostock IC Alberu J Arvizu A Hernández-Mendez EA De-Santiago A González-Tableros N. Probability of deceased donor kidney transplantation based on % PRA. Transpl Immunol (2013) 28(4):154–58. doi: 10.1016/j.trim.2013.05.002 23684945

[26] Jordan SC Pescovitz MD. Presensitization: the problem and its management. Clin J Am Soc Nephrol (2006) 1:421–32. doi: 10.2215/CJN.01651105 17699241

[27] Nimmo AMSA McIntyre S Turner DM Henderson LK Battle RK. The impact of withdrawal of maintenance immunosuppression and graft nephrectomy on HLA sensitization and calculated chance of future transplant. Transplant Direct (2018) 4(12):e409. doi: 10.1097/TXD.0000000000000848 30584590 PMC6283087

[28] Augustine JJ Woodside KJ Padiyar A Sanchez EQ Hricik DE Schulak JA. Independent of nephrectomy, weaning immunosuppression leads to late sensitization after kidney transplant failure. Transplantation (2012) 94(7):738–43. doi: 10.1097/TP.0b013e3182612921 22955228

[29] Del Bello A Congy-Jolivet N Sallusto F Guilbeau-Frugier C Cardeau-Desangles I Fort M. Donor-specific antibodies after ceasing immunosuppressive therapy, with or without an allograft nephrectomy. Clin J Am Soc Nephrol (2012) 7(8):1310–9. doi: 10.2215/CJN.00260112 PMC3408115 22626959

[30] Scornik JC Meier-Kriesche H. Human leukocyte antigen sensitization after transplant loss: timing of antibody detection and implications for prevention. Hum Immuno (2011) 72(5):398–401. doi: 10.1016/j.humimm.2011.02.018 21354453

[31] Ryu H Kim YC Moon JJ Song EY Min SI Ha J. Weaning immunosuppressant in patients with failing kidney grafts and the outcomes: a single-center retrospective cohort study. Sci Rep (2020) 10:6425. doi: 10.1038/s41598-020-63266-3 32286398 PMC7156393

[32] Lucisano G Brookes P Santos-Nunez E Firmin N Gunby N Hassan S. Allosensitization after transplant failure: the role of graft nephrectomy and immunosuppression – a retrospective study. Trans Int (2019) 32:949–59. doi: 10.1111/tri.13442 30980556

[33] Ayus JC Achinger SG Lee S Sayegh MH Go AS. Transplant nephrecotmy improves survival following a failed renal allograft. J Am Soc Nephrol (2010) 21(2):374–80. doi: 10.1681/ASN.2009050480 PMC2834539 19875809

[34] Leffell MS Kim D Vega RM Zachary AA Petersen J Hart JM. Red blood cell transfusions and the risk of allosensitization in patients awaiting primary kidney transplantation. Transplantation (2014) 97(5):525–33. doi: 10.1097/01.tp.0000437435.19980.8f 24300013

[35] Lubetzky M Tantisattamo E Molnar MZ Lentine KL Basu A Parsons RF. The failing kidney allograft: a review and recommendations for the care and management of a complex group of patients. Am J Transpl (2021) 21(9):2937–49. doi: 10.1111/ajt.16717 34115439

[36] Sapiertein Silva JF Fernandes Ferreira G Perosa M Nga HS de Andrade LGM. A machine learning prediction model for waiting time to kidney transplant. PloS One (2021) 16(5):e0252069. doi: 10.1371/journal.pone.0252069 34015020 PMC8136711

[37] Bayliss GP Gohh RY Morrissey PE Rodrigue JR Mandelbrot DA. Immunosuppression after renal allograft failure: a survey of US practices. Clin Transplant (2013) 27(6):895–900. doi: 10.1111/ctr.12254 24118389

[38] Alhamad T Lubetzky M Lentine KL Edusei E Parsons R Pavlakis M Kidney recipients with allograft failure, transition of kidney care (KRAFT): a survey of contemporary practices of transplant providers [published online ahead of print 2021] Am J Transplant (2021) 21(9):3034–42. doi: 10.1111/ajt.16523 33559315

[39] Casey MJ Wen Z Kayler LK Aiyer R Scornik JC Meier-Kriesche HU. Prolong immunosuppression preserves nonsensitization status after kidney transplant failure. Transplantation (2014) 98(3):306–11. doi: 10.1097/TP.0000000000000057 PMC4122584 24717218

[40] Park WY Paek JH Jin K Park SB Han S. Clinical significance of mycophenolate mofetil withdrawal in kidney transplant recipients. Trans Proc (2019) 51:2633–6. doi: 10.1016/j.transproceed.2019.03.061 31447192

[41] Ali AK Guo J Ahn H Shuster J. Outcomes of late corticosteroid withdrawal after renal transplantation in patients exposed to tacrolimus and/or mycophenolate mofetil: meta-analysis of randomized controlled trials. Int J Organ Transplant Med (2011) 2(4):149–59. PMC4089266 25013608

[42] Hricik DE Formica RN Nickerson P Rush D Fairchild RL Poggio ED. Adverse outcomes of tacrolimus withdrawal in immune-quiescent kidney transplant recipients. J Am Soc Nephrol (2015) 26(12):3114–22. doi: 10.1681/ASN.2014121234 PMC4657844 25925687

[43] Budde K Prashar R Haller H Rial MC Kamar N Agarwal A. Conversion from calcineurin inhibitor – to belatcept-based maintenance immunosuppression in renal transplant recipients: a randominzed phase 3b trial. J Am Soc Nephrol (2021) 32(12):3252–64. doi: 10.1681/ASN.2021050628 PMC8638403 34706967

[44] Karadkhele G Hogan J Magua W Zhang W Badell IR Mehta A. CMV high-risk status and posttransplant outcomes in kidney transplant recipients treated with belatacept. Am J Trans (2021) 21(1):208–21. doi: 10.1111/ajt.16132 32519434

[45] Schinstock C Tambur A Stegall M. Current approaches to desensitization in solid organ transplantation. Front Immunol (2021) 12:686271. doi: 10.3389/fimmu.2021.686271 34046044 PMC8144637

[46] Hickey MJ Zheng Y Valenzuela N Zhang Q Krystal C Lum E. New priorities: analysis of the new kidney allocation system on UCLA patients transplanted from the deceased donor waitlist. Hum Immunol (2017) 78(1):41–8. doi: 10.1016/j.humimm.2016.10.020 27818166

[47] Hart A Smith JM Skeans MA Gustafson SK Stewart DE Cherikh WS. OPTN/SRTR 2015 annual data Report:Kidney. Am J Transplant (2017) suppl 1):21–116. doi: 10.1111/ajt.14124 PMC5527691 28052609

[48] Loupy A Lefaucheur C Vernerey D Prugger C Duong van Huyen JP Mooney N. Complement-binding anti-HLA antibodies and kidney-allograft survival. N Engl J Med (2013) 369:1215–26. doi: 10.1056/NEJMoa1302506 24066742

[49] Tyan DB. Application, technical issues, and interpretation of C1q for graft outcome. Curr Opin Organ Transplant (2017) 22(5):505–10. doi: 10.1097/MOT.0000000000000454 PMC5662152 28723698

[50] Tambur AR Wiebe C. HLA diagnostics: evaluating DSA strength by titration. Transplantation (2018) 102(1S Suppl 1):S23–30. doi: 10.1097/TP.0000000000001817 29266059

[51] Tambur AR Campbell P Chong AS Feng S Ford ML Gebel H. Sensitization in transplantation: assessment of risk (STAR) 2019 working group meeting report. Am J Transplant (2020) 20:2652–68. doi: 10.1111/ajt.15937 PMC7586936 32342639

[52] Bu L Gupta G Pai A Anand S Stites E Moinuddin I. Clinical outcomes from the assessing donor-derived cell-free DNA monitoring insights of kidney allografts with longitudinal surveillance (ADMIRAL) study. Kid Int (2022) 101(4):793–803. doi: 10.1016/j.kint.2021.11.034 34953773

